# A phase I study of high-dose rosuvastatin with standard dose erlotinib in patients with advanced solid malignancies

**DOI:** 10.1186/s12967-016-0836-6

**Published:** 2016-03-31

**Authors:** Glenwood D. Goss, Derek J. Jonker, Scott A. Laurie, Johanne I. Weberpals, Amit M. Oza, Johanna N. Spaans, Charles la Porte, Jim Dimitroulakos

**Affiliations:** Ottawa Hospital Research Institute, Centre for Cancer Therapeutics, Ottawa, Canada; The Ottawa Hospital Cancer Centre, Ottawa, Canada; Department of Medicine, University of Ottawa, Ottawa, Canada; Division of Gynecologic Oncology, The Ottawa Hospital, Ottawa, Canada; University Health Network, Ontario Cancer Institute, Princess Margaret Hospital, Toronto, Canada; Faculty of Medicine and the Department of Biochemistry, University of Ottawa, Ottawa, Canada

**Keywords:** Statins, Pharmacokinetics, Epidermal growth factor receptor, Erlotinib, Therapeutics

## Abstract

**Background:**

Synergistic cytotoxicity with high-dose statins and erlotinib has been demonstrated in preclinical models across a number of tumour types. In this phase I study, we evaluated the safety and potential anti-tumour activity of escalating doses of rosuvastatin in combination with the standard clinical dose of erlotinib in heavily pretreated patients with advanced solid tumours.

**Methods:**

This was a single-center, phase I open-label study to determine the safety and recommended phase two dose (RPTD) of rosuvastatin in combination with 150 mg/day standard dose of erlotinib. Using a 3 + 3 study design and 28-day cycle, escalating doses of rosuvastatin from 1 to 8 mg/kg/day ×2 weeks (cycle 1) and 3 weeks (subsequent cycles) given concurrently with erlotinib were evaluated. In order to expand the experience and to gain additional safety and pharmacokinetic data, two expansions cohorts using concurrent or alternating weekly dosing regimens at the RPTD were also evaluated.

**Results:**

All 24 patients enrolled were evaluable for toxicity, and 22 for response. The dose-limiting toxicity (DLT) of reversible muscle toxicity was seen at the 2 mg/kg/day dose level. Maximal tolerated dose (MTD) was determined to be 1 mg/kg/day. Thirty-three percent of patients developed at least 1≥ grade 2 muscle toxicity (rhabdomyolysis: 1/24, myalgia: 7/24) resulting in one study-related death. Durable stable disease for more than 170 days was seen in 25 % of patients that received concurrent treatment and were evaluable for response (n = 16). Plasma erlotinib levels on study were unaffected by the addition of rosuvastatin.

**Conclusions:**

The observed disease stabilization rate of 25 % with combination therapy in this heavily pretreated population is encouraging, however, the high levels of muscle toxicities observed limited this combination strategy.

**Electronic supplementary material:**

The online version of this article (doi:10.1186/s12967-016-0836-6) contains supplementary material, which is available to authorized users.

## Background

A large body of experimental and clinical evidence supports the view that the epidermal growth factor receptor (EGFR) is a relevant target for cancer therapy [1–3]. Two small molecule EGFR tyrosine kinase inhibitors (EGFR-TKIs; gefitinib and erlotinib) are approved for the treatment of advanced non-small cell lung cancer (NSCLC) in the first-line setting in patients whose tumors harbor an activating EGFR mutation [4, 5]. Despite early response rates of 60–80 %, resistance to EGFR-TKIs in this population usually develops within a year [6–9]. EGFR-TKIs are also approved as second-line therapy in unselected NSCLC patients who have failed chemotherapy, with response rates of 10–12 % and improved overall survival of 2 months, from 4.7 to 6.7 months [10]. In other epithelial derived cancers that generally express wild-type EGFR like squamous cell carcinomas, breast and prostate cancers, response rates to EGFR-TKIs are generally less than 5 % [11–13]. Given its modest single agent activity in the wild-type population, novel combination regimens with EGFR-TKIs are required.

Mevalonate pathway metabolites are critical for the function or the appropriate expression/localization of receptor tyrosine kinases (RTKs), including EGFR, and the effectors of their downstream signaling cascades [14–16]. The rate-limiting step of the mevalonate pathway is the conversion of HMG-CoA to mevalonate, which is catalyzed by HMG-CoA reductase [15]. Deregulated or elevated activity of HMG-CoA reductase has been demonstrated in a variety of different tumours [17, 18]. The statin family of drugs are potent inhibitors of HMG-CoA reductase that are well-tolerated and widely used as treatments for hypercholesterolemia at standard daily doses of 5–40 mg/day [17] with the most common side effects being myalgia, nausea, diarrhea and constipation [19]. Importantly, in preclinical studies high-dose statin treatment has been shown to directly block tumour cell growth, invasion and metastatic potential both in vitro and in vivo [18, 20, 21]. In a phase I study of lovastatin monotherapy in patients with solid tumors (n = 88), the MTD was determined to be 25 mg/kg/day based on 1-week of consecutive dosing, followed by a 3-week break every 28-day cycle [22]. Although roughly 25 times the normal upper limit of doses used to treat hypercholesterolemia, this weekly regimen failed to demonstrate clinical efficacy as an anticancer therapy [22]. Of importance to this study, we demonstrated that HMG-CoA reductase was a target of retinoid action [23] and that retinoid responsive tumour types are particularly sensitive to statin-induced apoptosis that includes acute myeloid leukemias, paediatric malignancies and squamous cell carcinomas (SCC) [24, 25]. This work led to our phase I trial in recurrent SCC (n = 26) using a prolonged oral administration of lovastatin that established 7.5 mg/kg/day × 21 days in a 28-day cycle as the MTD, with a DLT of reversible muscle toxicity [26]. Importantly, in this study 23 % of these end-stage patients displayed disease stabilization lasting greater than 3 months, albeit in the absence of any objective responses [26]. With the aim to enhance their clinical anticancer efficacy, high-dose statins where then evaluated in combination with EGFR-TKIs, as detailed below.

In preclinical studies, we demonstrated that combination treatment with lovastatin and EGFR-TKIs at concentrations between 1 and 10 μM induced synergistic cytotoxicity in SCC, NSCLC and colon cancer cell lines [27, 28]. Statin treatment was shown to inhibit ligand induced dimerization, autophosphorylation and intracellular trafficking of the EGFR [29]. Based on this encouraging preclinical data and given the manageable toxicities of high-dose single-agent statin observed in prior phase I studies [22, 26], we undertook this current phase I study, evaluating escalating doses of rosuvastatin with standard dose erlotinib. Erlotinib was chosen based on its safety and tolerability profile in phase I–III studies, the most common side-effects consisting of rash, fatigue, anorexia and diarrhea [30]. Rosuvastatin was chosen for its enhanced bioavailability and activity over lovastatin [31]. Further, as a non-CYP3A4 substrate [32], rosuvastatin was expected to have a reduced likelihood of drug:drug interaction with erlotinib at the elevated doses required to achieve relevant anticancer serum levels. The specific aims of this phase I study were to define the RPTD, DLTs and the pharmacokinetic interaction of rosuvastatin with erlotinib when used in combination in the treatment of patients with advanced solid tumours.

## Methods

### Study design and eligibility

This was a single-center, phase I open-label study to determine the safety and RPTD of rosuvastatin in combination with the standard clinical dose of erlotinib (150 mg/day) in patients with advanced solid tumours. This study was approved by Health Canada and the Ottawa Hospital Research Ethics Board (2007908-01H) and described at ClinicalTrials.gov (Identifier: NCT00966472). Eight cohorts from 1 to 8 mg/kg/day rosuvastatin given concurrently with standard dose erlotinb in 28-day cycles were planned. Eligible adult patients had a diagnosis of advanced/metastatic (stage IIIB/IV) incurable solid malignancies. Patients received therapy until evidence of disease progression, the development of serious or unmanageable adverse events (SAE), or until study withdrawal. Clinically or radiological documented disease and an ECOG performance status of 0, 1 or 2 was also required. All study patients had adequate hematogical, hepatic and renal functions at baseline. Adequate hematological function was defined as hemoglobin ≥90 g/L, absolute granulocyte count ≥1.5 × 10^9^/L and platelet counts ≥100 × 10^9^/L. Biochemical parameters for hepatic function included; total bilirubin <1.5× the upper limit of normal (ULN) and ALT/AST <1.5× ULN. Adequate renal function was defined as a creatinine clearance >60 ml/min as measured by 24 h urine collection. Any anticholesterol agents were discontinued at least 7 days prior to starting the trial. Other exclusion criteria included known brain or leptomeningeal metastases, no prior EGFR inhibitor therapy, no known neuromuscular disorders as well as known hypersensitivity or allergy to HMG-CoA reductase inhibitors including rosuvastatin in Asian populations which were also excluded [33], (US FDA Public Health Advisory, 2 March 2005, www.fda.gov).

### Treatment

Erlotinib was provided by Roche Canada and was administered orally in tablet form at a standard daily dose of 150 mg. This dose was chosen based on its established safety and efficacy in pre-treated patients with advanced cancer [30]. The starting dose of rosuvastatin was 1 mg/kg/day with the dose of rosuvastatin increasing in a step-wise manner in escalating doses of 1 mg/kg/day. The 1 mg/kg/day starting dose (~80 mg/day) represents the highest tested hypercholesterolemia dose for rosuvastatin and was chosen for safety [34]. During cycle-1 of the dose escalation, treatment with rosuvastin was administered as follows: 1 week off, then daily for 2 weeks, then 1 week off. For all subsequent cycles, rosuvastatin was given daily for the first 3 weeks of each 28-day cycle, a schedule that was successfully employed in the previous phase I study of lovastatin in SCC patients [26]. Doses were rounded to the nearest 5 mg since rosuvastatin is available in 5, 10, 20 and 40 mg tablets (purchased from AstraZeneca, Canada). Oral administration of rosuvastatin was once daily if daily dose was <80 mg, twice if 81–160 mg, three times a day if 161–240 mg and four times a day if >240 mg daily dose. Similar dosing schedules were incorporated in previous high dose statin trials [22, 26]. No prophylactic medications were supplied. Standard supportive care medications were allowed. Oral supplementation of ubiquinone at 60 mg po QID was initiated if patient developed grade 3 or 4 muscle toxicity and continued until recovery to baseline.

A 3 + 3 dose escalation phase I study design was used, with 3 patients planned at each cohort. If no dose-limiting toxicity during the first treatment cycle was observed in the first 3 patients, then dose escalation was planned for the next cohort of 3 patients. All 3 patients at a given dose level needed to complete the first cycle of treatment without suffering DLT before new patients were enrolled at the next dose level. The lead patient at each new dose level completed the first cycle of treatment before other patients were enrolled at the same dose level. If 1/3 initial patients at each dose experienced a DLT, then the treatment level was expanded to at least 6 patients. If no more than 1 of 6 patients experienced DLT, then the next cohort of patients was to be treated at the next higher dose level. There was no dose escalations allowed within individual patients. If >2 patients at any dose level experienced DLT, then that level was considered to have exceeded the maximal tolerated dose (MTD), and the level immediately preceding that level was designated as MTD and RPTD. An expansion cohort was also proposed at the RPTD of rosuvastatin to expand experience and to gain additional safety and efficacy data of this combination regimen.

### Toxicity

All toxicity grading was according to the NCI common toxicity criteria version 2 [35]. DLT was defined as any first-course, >grade 3 non-hematological toxicity (excluding alopecia or inadequately controlled nausea/vomiting), grade 4 neutropenia or with fever, grade 4 thromocytopenia, or dose delay of >2 weeks due to drug-related toxicity. Patients who recovered from DLT were able to continue on study with a dose reduction to the next lower dose level at investigator’s discretion.

### On study evaluation

Patients had weekly laboratory evaluations for hematogical, hepatic and renal functions and biochemical changes including creatine and creatine phosphokinase (CPK) and fasting total cholesterol, HDL and LDL during cycle 1 and at the start of each subsequent cycle. Physical examinations occurred at the start of each cycle and radiological tumor evaluations were completed after every 2 cycles. Tumor response was assessed using response evaluation criteria in solid tumors (RECIST) criteria [36].

### Treatment duration and follow-up

All patients received treatment until clinical and/or radiologic progression, unacceptable toxicity, patient refusal or if the treating physician felt that continued participation was no longer in the best interest of the patient. In these cases, the patient went off protocol treatment. Patients with objective response, disease stabilization, or symptomatic improvement were allowed to continue the treatment at the investigator’s discretion until evidence of disease progression or serious toxicity. Myalgia, myopathy and, rarely rhabdomyolysis may occur in patients treated with rosuvastatin at all doses [34] presenting with muscle pain or muscle weakness in conjunction with increases in creatine kinase (CPK) values to greater than 10 times the upper limit of normal. For Grade 3 events, rosuvastatin treatment will be held until resolution and restarted at one lower dose level and for Grade 4 toxicities; the patient will be removed from the protocol. Similar strategies were employed for other toxicities as well. All patients were seen in follow-up 4 weeks after the last treatment and were monitored for any on-going adverse effects, late adverse effects and death. Institutional Research Ethics Board approval was obtained prior to the start of this study, and all study subjects provided written informed consent. This study was conducted according to Good Clinical Practice.

### Pharmacokinetic evaluation and analysis

Unlike other statins, rosuvastatin is not a CYP3A4 substrate [33] and was not expected to display drug:drug interaction with erlotinib. However, due to the significantly higher doses of statins required to achieve anticancer serum levels, we undertook pharmacokinetics of this treatment combination. Pharmacokinetic analysis of erlotinib serum levels was undertaken at day 6 (erlotinib alone) and at day 14 (erlotinib and 1 mg/kg/day rosuvastatin treatments) and rosuvastatin at the day 14 timepoint of cycle 1 in 6 study patients (Schedule A). On day 6 and 14 serum erlotinib levels and day 14 serum rosuvastatin levels were determined at 2 h intervals for a full 24 h after drug treatments. The analysis of rosuvastatin and erlotinib was performed by a validated liquid chromatography mass spectrometry/mass spectrometry (LC/MS/MS) method [37, 38]. Protein precipitation (plasma: 50/50 acetonitrile/menthol 1:2) was used for sample preparation before injecting to HPLC for separation. Rosuvastatin, erlotinib and internal standard (IS), 6,7-Dimethyl-2,3-di(2-pyridyl)-quinoxaline (DMDPQ) was eluted by HPLC (Accela, Thermo) using a gradient mobile phase [0.2 % Formic acid (V:V), and 100 % methanol]. The chromatographic separation was achieved at room temperature on a 1.9 μm C18 column (Hypersil Cold; 2.1 × 50 mm, Thermo) equipped with a 2-cm pre-column packed with the same material at 200 μL/min. Detection was done by tandem Mass spectrometer (TSQ Quantum Access MAX, Thermo) with electrospray ionization in positive mode. The multiple reaction monitoring (MRM) was be used to detect specific precursor ion to product ion transitions for rosuvastatin (m/s 482.3/258.1), erlotinib (m/s 394.3/336.2), and IS (m/s 313.2/91.2), respectively. Xcalibur and TSQ Tune master software (Thermo) was used as the system controller and integrator.

Both rosuvastatin and erlotinib were stable for 24 h at 4 °C after sample preparation and during 3 freeze-thaw cycles. The linear calibration ranges in plasma were from 0.25 to 200 μg/L for Rosuvastatin and 6.25–3000 μg/L for Erlotinib, respectively. The recoveries were above 85 % for both drugs at three concentrations. And Intra- and inter-day precisions for both drugs were less than 12 %.

## Results

### Study conduct

**Fig. 1:**
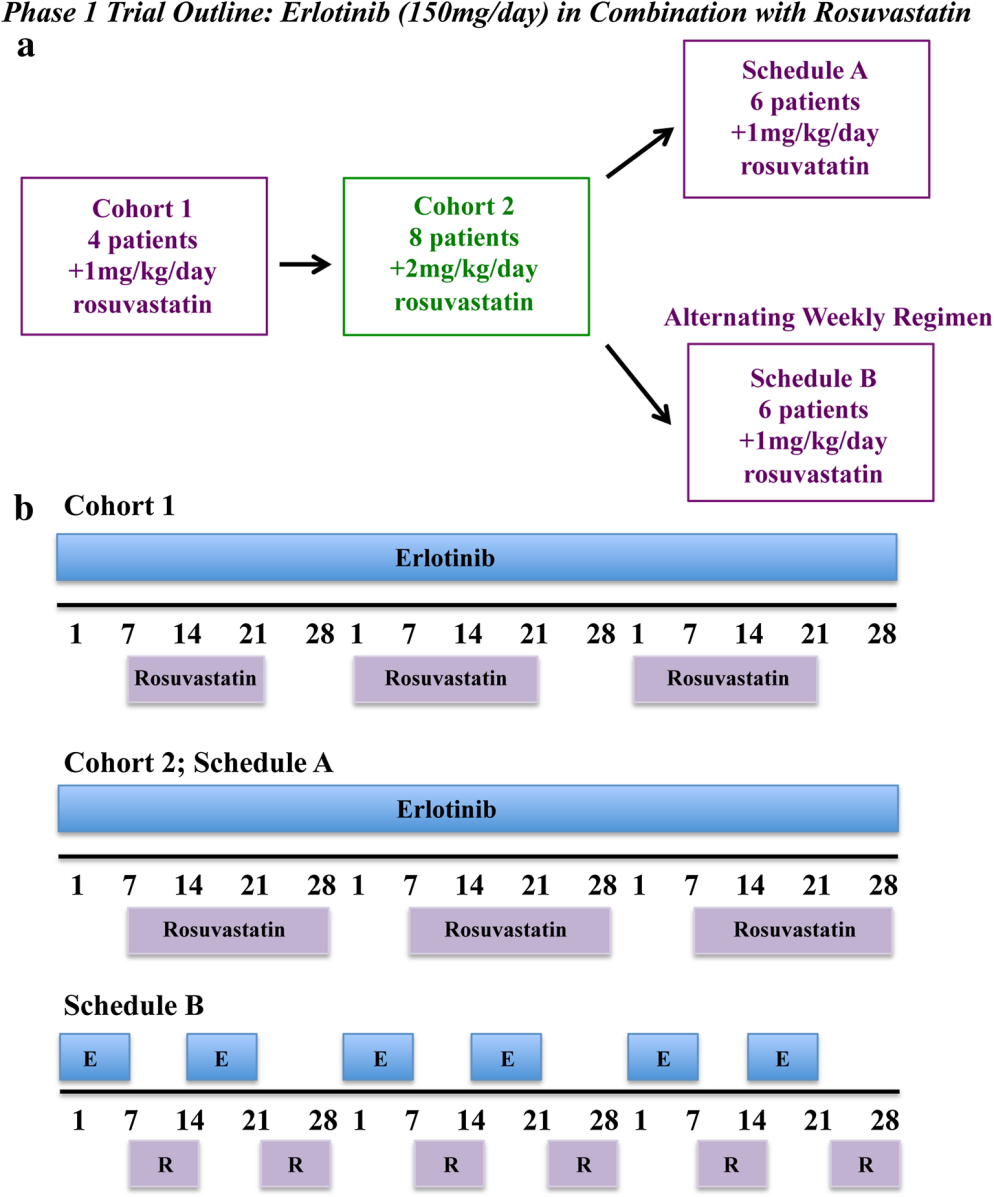
Phase I trial outline. **a** Schematic of the patient cohorts including patient numbers and treatment regimens evaluated in this study. **b** Treatment schedules employed in this Phase I study

Two levels of dose-escalation were attempted. One of the last patients enrolled in the second cohort (2 mg/kg/day rosuvastatin) developed Grade 4 rhabdomyolysis, which led to a study related death and established the 1 mg/kg/day dose of rosuvastatin as the MTD and RPTD when used in combination with 150 mg of erlotinib. As a result of this unexpected death, the study was amended to include a second expansion cohort at the RPTD to evaluate the impact of alternating weekly erlotinib and rosuvastatin treatment on the safety of this combination regimen. As detailed in Fig. 1, four patients were enrolled at the first dose (1 mg/kg/day) as the second study patient withdrew consent prior to receiving rosuvastatin and eight patients were enrolled in the second cohort (2 mg/kg/day). At the 2 mg/kg/day escalation dose, 8 patients were enrolled with the original 3 patients to be expanded by another 6 patients due to the development of skin rash in the 3 patients that is associated with erlotinib clinical activity but stopped at a total of 8 patients due to a study related death. An additional 12 patients were accrued to the expansion cohorts, with 6 patients enrolled to into Schedule A (an expansion of Cohort 1 at 1 mg/kg/day rosuvastatin) and 6 patients into Schedule B, which used an alternating weekly schedule of rosuvastatin (1 mg/kg/day) and erlotinib (Fig. 1b). In total, eighteen patients were treated concurrently with erlotinib and rosuvastatin and six with an alternating weekly schedule of rosuvastatin and erlotinib.

### Patient characteristics

Patient characteristics are outlined in Table 1, according to the type of combination regimen received (concurrent or alternating). There were similar median ages and gender ratios between the concurrent treatment group (Cohorts 1, 2 and Schedule A) and the alternating treatment group (Schedule B) at 60 vs 54 years and 60 vs 67 % male gender, respectively. A variety of tumor types were represented in both the concurrent and alternating treatment regimens including NSCLC, which represented 29 % of all patients evaluated. Patients were generally heavily pre-treated, with 70 % having undergone at least two previous treatment regimens and 80 % having received prior radiation therapy.

**Table 1:** Patient characteristics

	Concurrent Cohorts 1 and 2/Schedule A	Alternating Schedule B	All patients
Number of patients	18	6	24
Age (years)			
Median/range	60/43–70	54/44–63	58/43–70
Gender			
Male	11 (60 %)	4 (67 %)	15 (63 %)
Female	7 (40 %)	2 (33 %)	9 (37 %)
Ethnicity			
Caucasian			23 (96 %)
Other			1 (4 %)
Primary tumour			
NSCLC	5	2	7
Esophageal	4		4
Pancreas	2	1	3
GU	4	2	6
Colon	2		2
Breast	1		1
Unknown		1	1

Number	Frequency	Percent
Previous treatment regimens		
1	7	29.2
2	8	33.3
3	6	25.0
>3	3	12.5
Reason Off protocol		
Progressive disease	19	79.2
Adverse event	1	4.2
Patient withdrawal	2	8.3
Other	2	8.3

### Response

**Fig. 2:**
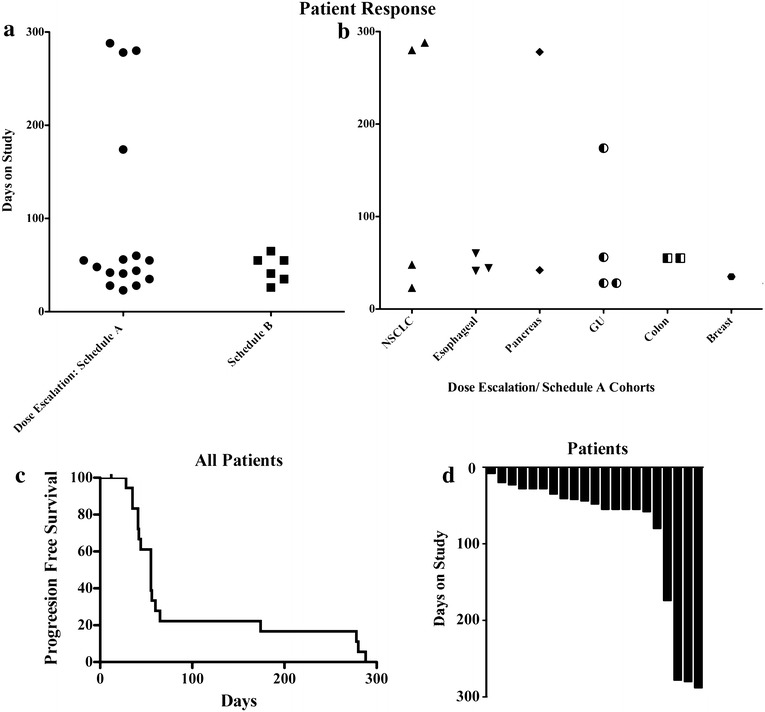
Patient responses on study. **a** Days on study for each evaluable patient segregated into concurrent treatments (Cohorts 1 and 2-dose escalation and Schedule A) and the alternating weekly schedule (Schedule B). **b** Days on study based on tumour type in the concurrent treatments. **c** Kaplan-Meir curve evaluating progression free survival in all evaluable patients. **d** Waterfall plot of the number of days on study of evaluable patients in this study

Twenty-four eligible patients were enrolled on study with two patients not evaluable for response due to early study withdrawal during the first cycle. In addition to these two patients, another five patients did not complete cycle 1 due to progressive disease. No objective responses were seen employing RECIST criteria [36]. Patients were analyzed based on whether they received concurrent treatments with these two agents (Cohorts 1 and 2 and Schedule A, a total of 16 evaluable patients) or were treated with alternating weekly does of rosuvastatin and erlotinib treatments (Schedule B, 6 evaluable patients). Of particular interest, 4/16 evaluable patients treated concurrently were on study for greater than 170 days, with 3 patients on study for greater than 275 days (Fig. 2a). The tumour types of patients displaying durable stable disease were 2/4 NSCLC patients, 1/2 pancreatic cancer (responsive patient; neuroendocrine tumour) and 1/4-genitourinary (GU) cancer patients (vaginal squamous epithelial carcinoma) (Fig. 2b). This stable disease is highlighted by the Thorax CT scans of one of the NSCLC patients that was on study, the pre-treatment scan at-15 days and a subsequent scan at 218 days on study are shown (Additional file 1: Figure S1). Further, of the patients that came off study with progressive disease (n = 19), a subset of these patients also displayed prolonged progression free survival of greater than 175 days (Fig. 2c) and a waterfall plot of patient days on study also highlight the subset of patients with stable disease (Fig. 2d).

### Toxicities

All 24-study patients were evaluable for toxicity. Adverse events were documented for each treatment regimen. The ≥Grade 2 toxicities are listed in Table 2 and are consistent with those previously observed with single agent erlotinib or rosuvastatin. Anorexia (15/24 patients) and rash (7/24), common toxicities observed with erlotinib treatment, were observed in all cohorts. Muscle toxicities (grade ≥2) known to be associated with statin therapy, included fatigue (18/24), muscle weakness (10/24) and myalgia (7/24) and were common across all cohorts. The rate of muscle pathologies was more pronounced at the 2 mg/kg/day dose, indicating the potential of a dose effect. At this higher dose, a single patient out of 8 evaluable patients developed rhabdomyolysis, which resulted in a study related death. This patient had baseline and day 6 (erlotinib only) serum levels in the normal range for alanine transaminase (ALT), albumin and CPK measures of hepatic, renal and muscle health, respectively. However at day 28 (erlotinib and rosuvastatin treatment), significant increases in all three-serum markers were observed, particularly with CPK levels which increased from 60 U/L to over 2000 U/L, suggesting rosuvastatin-induced muscle damage (Additional file 2: Figure S2). In the 2 mg/kg/day dose (Cohort 2), 4 out of the 8 patients enrolled had elevated CPK levels during rosuvastatin treatments compared with only 1 out of 16 patients at the 1 mg/kg/day dose. Surprisingly, treatment with rosuvastatin did not result in significant changes in serum cholesterol, LDL or HDL levels, even in patients on study for greater than 170 days (Additional file 3: Figure S3). Given the elevated CPK levels and the DLT of muscle toxicity observed at the 2/mg/kg/day dose level, 1 mg/kg/day was established as the RPTD.

**Table 2:** Adverse events

Patients	1 mg/kg/day	2 mg/kg/day	Alternating	All n = 24
	Cohort 1/Schedule A n = 10	Cohort 2 n = 8	Schedule B n = 6	
Completed 1st cycle	7/10 (70 %)	5/8 (63 %)	5/6 (83 %)	17/24 (71 %)

Toxicities grade (%)	2	3	4		2	3	4		2	3	4	
Rash	1				4	2						29.2
Diarrhea	3	1			1							20.8
Anorexia	5	2			4				4			62.5
Weight loss	1	1							2			16.7
Anxiety	1											4.2
Nausia	3				1							16.7
Vomiting	4											16.7
Fatigue	4	4			5	1			2	2		75.0
Muscle weakness	1	1			6				1	1		41.7
Myalgia		2			3	1			1			29.2
Rhabdomyolysis							*1*					4.2^a^

^a^Study related death

### Pharmacokinetic analyses

**Fig. 3:**
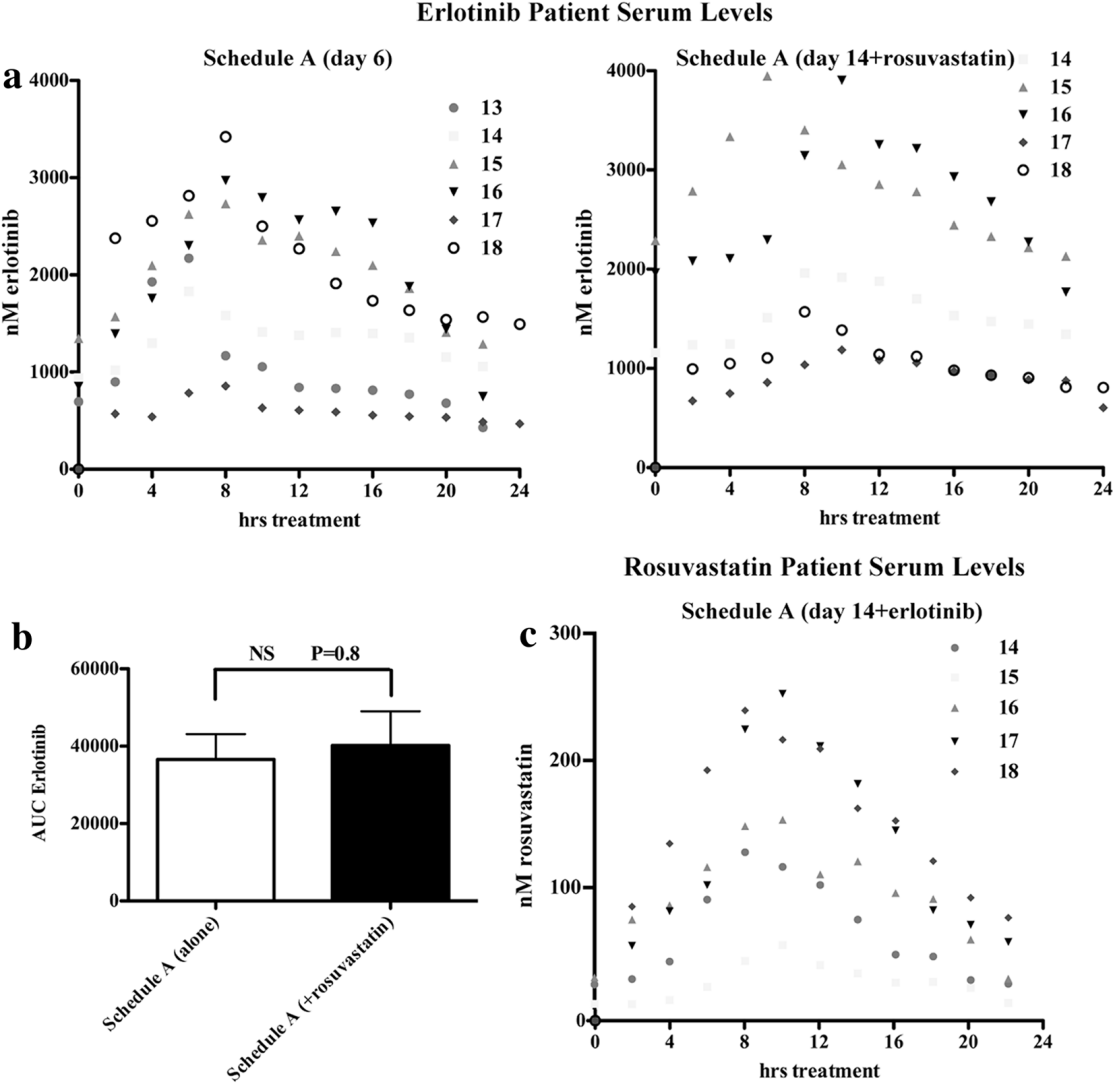
Pharmacokinetic analysis of Schedule A patients. **a** Serum erlotinib levels were determined for these patients at day 6 (erlotinib alone,* left panel*) and at day 14 (erlotinib + rosuvastatin treatments) every 2 h following drug administration for 24 h. **b** For the 5 evaluable patients (14–18), area under the curve (AUC) values showed no statistical difference between the two treatments with respect to serum erlotinib exposure. P value determined by *T* test. **c** Serum rosuvastatin levels were determined for these patients at day 14 (erlotinib +1 mg/mg/day rosuvastatin treatments) every 2 h following drug administration for 24 h

Pharmacokinetic analysis of erlotinib serum levels was performed at day 6 (erlotinib alone) and at day 14 (erlotinib and 1 mg/kg/day rosuvastatin treatments) in the 6 patients enrolled in Schedule A (Fig. 1). The erlotinib levels at each time-point for each patient are depicted in Fig. 3a. In the five evaluable patients who received both erlotinib and rosuvastatin, the area under the curve (AUC) for erlotinib did not differ significantly between day 6 and day 14 (Fig. 3b). Furthermore, the maximum concentration observed for each patient (Cmax) and the time to obtain the Cmax (Tmax) were also similar in both treatments (Table 3). The half-life of erlotinib was also consistent and was unaffected by the addition of rosuvastatin. Pharmacokinetic analysis of rosuvastatin serum levels was also performed at day 14 (erlotinib and 1 mg/kg/day rosuvastatin treatments; single dose) in the five evaluable patients who received both erlotinib and rosuvastatin, the area under the curve (AUC) for rosuvastatin is presented (Fig. 3c). As expected the nM serum concentrations are elevated compared to the hypercholesterolemia dose (approximately 1/2 to 1/4 the dose employed in this study) [39]. Patient variability has been consistently demonstrated irrespective of dose [39] and is observed in this study as well, however, due to the treatment schedule employed a comparison in the same patients of rosuvastatin serum levels with or without erlotinib treatment were not evaluated.

**Table 3:** Pharmacokinetic analysis of erlotinib in Schedule A patients

	13	14	15	16	17	18
Erlotinib alone (day 6)						
AUC (μM)	23.45	29.68	45.44	46.19	13.87	50.14
Cmax (μM)	2.17	1.83	2.73	2.97	0.86	3.42
Tmax (h)	6	6	8	8	8	8
t _1/2_ (h)	7.1	25.7	18.1	15.1	15.4	9.4
Erlotinib (+rosuvastatin) (day 14)						
AUC (μM)	off study	34.32	62.74	59.51	21.22	24.81
Cmax (μM)	off study	1.96	3.95	3.90	1.19	1.57
Tmax (h)	off study	8	6	10	10	8
t_1/2_ (h)	off study	24.3	14.5	12.8	12.8	13.3

## Discussion

In our phase I study of combination therapy with high-dose rosuvastatin and standard dose erlotinib, concurrent treatment resulted in stable disease lasting >170 days in 25 % of these heavily pretreated patients, albeit in the absence of objective responses. Despite choosing a drug combination that minimized the chance of drug:drug interactions, this combination regimen showed significant toxicities. The rate of myalgia in our study (34 %), was substantially higher than the rate observed with therapeutic doses of statin therapy used in the treatment of hyperlipidemia (1–5 %) [40]. Two escalating levels of rosuvastatin were performed, based on a standard 3 + 3 study design, with the second dose of rosuvastatin resulting in a study-related death.

The dose-limiting muscle toxicities associated with the combined administration of high-dose rosuvastatin and erlotnib was unexpected, and had not been previously reported in phase I studies with single agent high-dose statin treatment in solid tumours [22]. Notably, in our earlier phase I study of single agent lovastatin in SCC patients with advanced cancers of the head and neck or cervix, of the 24 patients enrolled, 5 patients experienced reversible dose-limiting muscle toxicities, only 2 of which were greater than grade 2 in severity [26]. The low μM concentration range (0.8–3.9) and intra-patient variability observed following 150 mg treatment with erlotinib is in keeping with previously published reports [41]. Our pharmacokinetic data suggest a lack of drug:drug interaction in patients treated concurrently with standard dose erlotinib and high-dose rosuvastatin, and cannot explain the excessive toxicity observed with this combination regimen. Given the absence of an observed drug:drug interaction, our results support the continued use of low dose rosuvastatin in the treatment of hypercholesterolemia in patients on erlotinib.

In unselected patients, high dose single agent statin treatments have not demonstrated clinical activity. In our phase I study in SCC patients, a tumour type that showed significant statin-induced apoptosis in vitro, disease stabilization of greater than 3 months in 23 % of patients was observed [26]. In this study, in a non-selected patient population that included a single SCC patient, similar rates but more pronounced disease stabilization (greater than 6 months for 4/16 patients in the concurrent treatment regimens) was observed. This included 2/4 NSCLC, 1/2 pancreatic cancer (responder; neuroendocrine tumour) and a single SCC (vaginal carcinoma) patient. In a recent study conducted by Han et al. [42], the hypercholesterolemia dose of simvastatin was employed in a daily regimen with the standard dose of gefitinib in NSCLC patients. In particular, SCC patients displayed higher response rates and longer PFS compared to gefitinib alone. This study was based on our work that showed in vitro synergy of this approach as well as the ability of statins to inhibit EGFR function [27, 29, 42, 43]. Taken together, these results suggest that high-dose statin therapy in combination with EGFR-TKIs may be beneficial in a subgroup of patients with advanced solid cancers.

Although contributing to our understanding of the therapeutic potential of high-dose statins as part of combination anti-cancer therapy, our study has limitations. Due to limited tumor availability, EGFR mutation testing was only possible on 8/24 study patients (all wild-type, including the SCC patient with stable disease). As such, EGFR mutation positivity cannot be discounted as a possible reason for the prolonged stable disease observed in the two NSCLC patients (mutation status unknown), although this response is not typical of the tumour regressions observed in erlotinib treated EGFR activating mutation patients [7]. Our study established 1 mg/kg/day (for 3/4 weeks) as the RPTD of rosuvastatin in combination with standard daily dosing of 150 mg/day erlotinib with a significant durable stable disease observed in a subgroup of our heavily pre-treated patients. Given the significant dose limiting muscle toxicities associated with their combined use, alternate treatment strategies that can mimic the anti-cancer effects of statins without the associated toxicities may represent a more refined therapeutic approach in the treatment of advanced cancer.

## Conclusions

Our laboratory in preclinical models has demonstrated synergistic cytotoxicity with high-dose statins and EGFR-TKIs across a number of tumour types. In this phase I study, we employed escalating doses of rosuvastatin in combination with erlotinib in patients with advanced solid tumours. Importantly, no drug:drug interactions were demonstrated between these two agents. The observed disease stabilization rate of 25 % with combination therapy in this heavily pretreated population is encouraging, however, the high levels of muscle toxicities observed limited this combination strategy.

## Additional files

10.1186/s12967-016-0836-6 Thorax computerized tomography (CT) scans of the NSCLC Patient 9 who exhibited durable stable disease. Pre-treatment scan at -15 days prior to enrollment is depicted with the corresponding scan at 218 days on study. Red arrow highlights the tumour mass.
10.1186/s12967-016-0836-6 Serum marker levels in Patient 11 who developed rhadbomyolysis on study. Baseline and Day 6 (erlotinib only) serum levels in the normal range for alanine transaminase (ALT), albumin and CPK, measures of hepatic, renal and muscle health, respectively. However at Day 28 (erlotinib and rosuvastatin treatment), significant increases in all three-serum markers were observed, particularly with CPK levels, which increased from 60U/L to over 2000U/L, suggesting rosuvastatin-induced muscle damage.
10.1186/s12967-016-0836-6 Serum cholesterol, low-density lipoprotein (LDL) and high-density lipoprotein (HDL) levels in the 4 patients with durable stable disease in this study. Their levels remained consistent throughout this study in all 4 patients.

## Abbreviations

RPTD: recommended phase two dose
DLT: dose-limiting toxicity
MTD: maximal tolerated dose
EGFR: epidermal growth factor receptor
EGFR-TKIs: EGFR tyrosine kinase inhibitors
RTKs: receptor tyrosine kinases
SCC: squamous cell carcinomas
NSCLC: non-small cell lung carcinomas
SAE: serious adverse events
CPK: creatine phosphokinase
RECIST: response evaluation criteria in solid tumors
GU: genitourinary
