# Supplementary figures and images for: A phase I study of high-dose rosuvastatin with standard dose erlotinib in patients with advanced solid malignancies
Source: J Transl Med. 2016 Mar 31;14:83. doi: 10.1186/s12967-016-0836-6 (PMC4815068; doi:10.1186/s12967-016-0836-6)

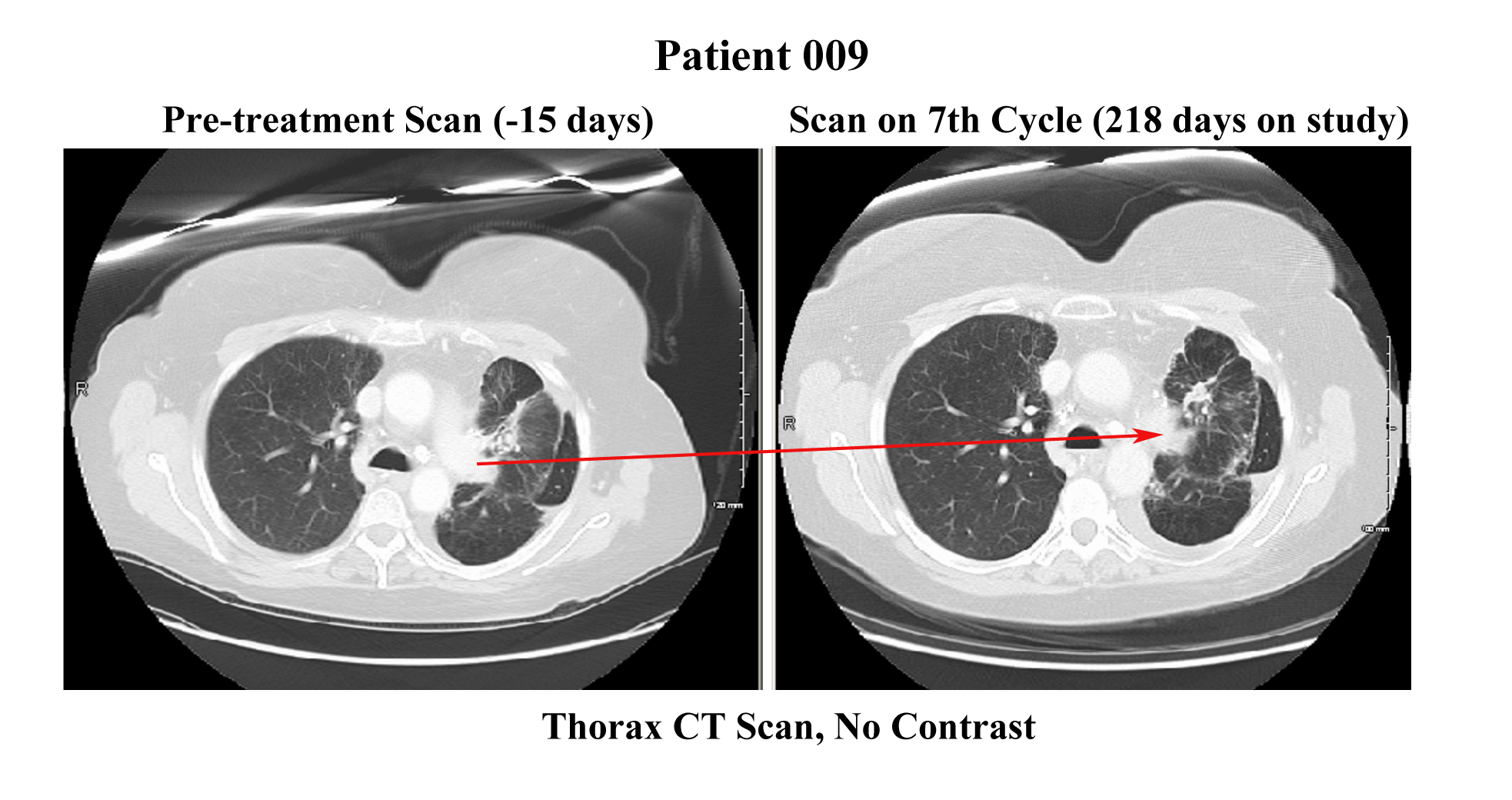

Supplement: Supplementary file 1 — 10.1186/s12967-016-0836-6 Thorax computerized tomography (CT) scans of the NSCLC Patient 9 who exhibited durable stable disease. Pre-treatment scan at -15 days prior to enrollment is depicted with the corresponding scan at 218 days on study. Red arrow highlights the tumour mass. [file 12967_2016_836_MOESM1_ESM.tif]

## Slide 1
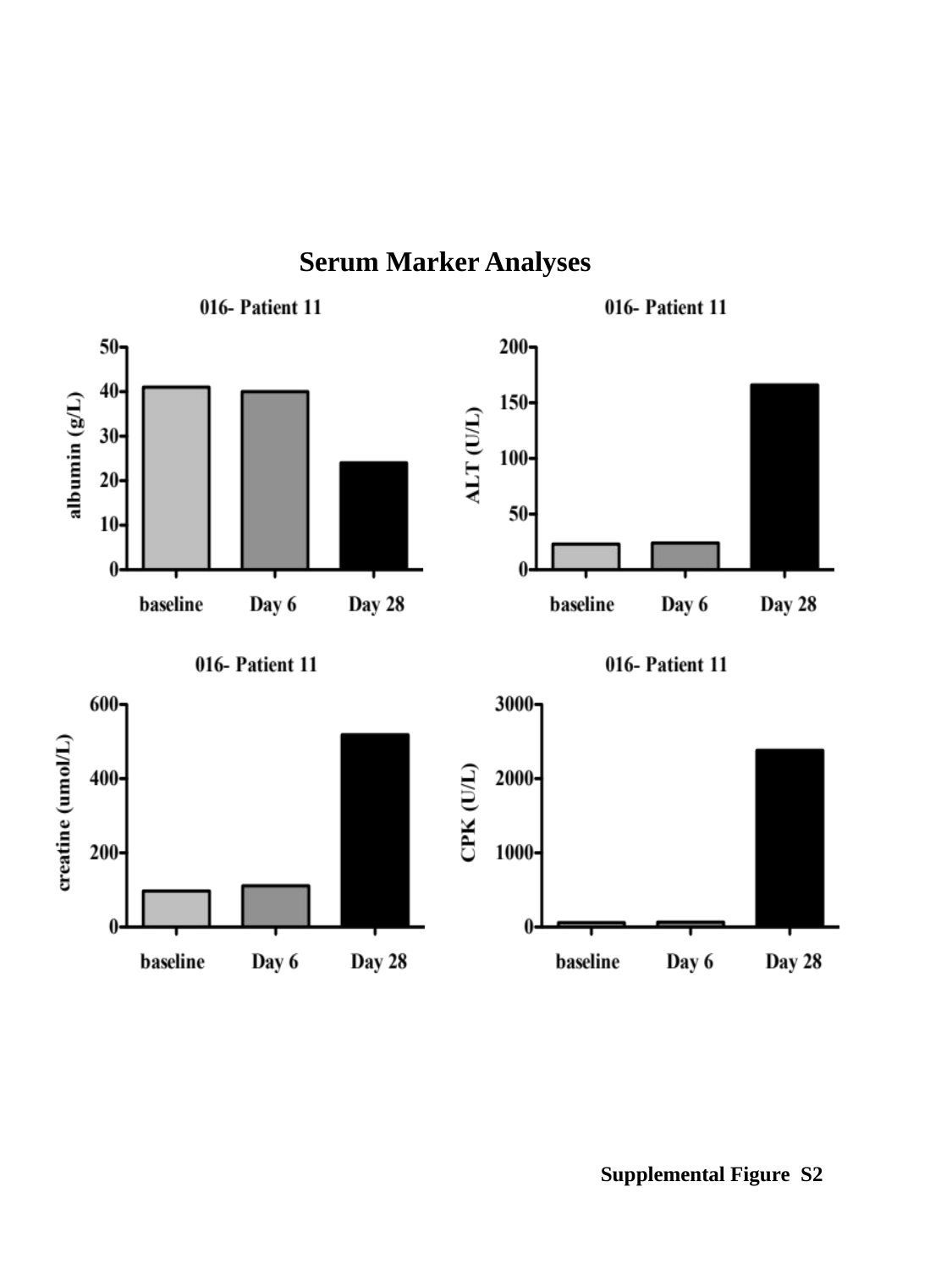

Serum Marker Analyses
Supplemental Figure S2

Supplement: Supplementary file 2 — 10.1186/s12967-016-0836-6 Serum marker levels in Patient 11 who developed rhadbomyolysis on study. Baseline and Day 6 (erlotinib only) serum levels in the normal range for alanine transaminase (ALT), albumin and CPK, measures of hepatic, renal and muscle health, respectively. However at Day 28 (erlotinib and rosuvastatin treatment), significant increases in all three-serum markers were observed, particularly with CPK levels, which increased from 60U/L to over 2000U/L, suggesting rosuvastatin-induced muscle damage. [file 12967_2016_836_MOESM2_ESM.pptx]
