# Supplementary material for: A phase I study of high-dose rosuvastatin with standard dose erlotinib in patients with advanced solid malignancies
Source: J Transl Med. 2016 Mar 31;14:83. doi: 10.1186/s12967-016-0836-6 (PMC4815068; doi:10.1186/s12967-016-0836-6)
Supplement: Supplementary file 3 — 10.1186/s12967-016-0836-6 Serum cholesterol, low-density lipoprotein (LDL) and high-density lipoprotein (HDL) levels in the 4 patients with durable stable disease in this study. Their levels remained consistent throughout this study in all 4 patients. [file 12967_2016_836_MOESM3_ESM.pptx]

## Slide 1
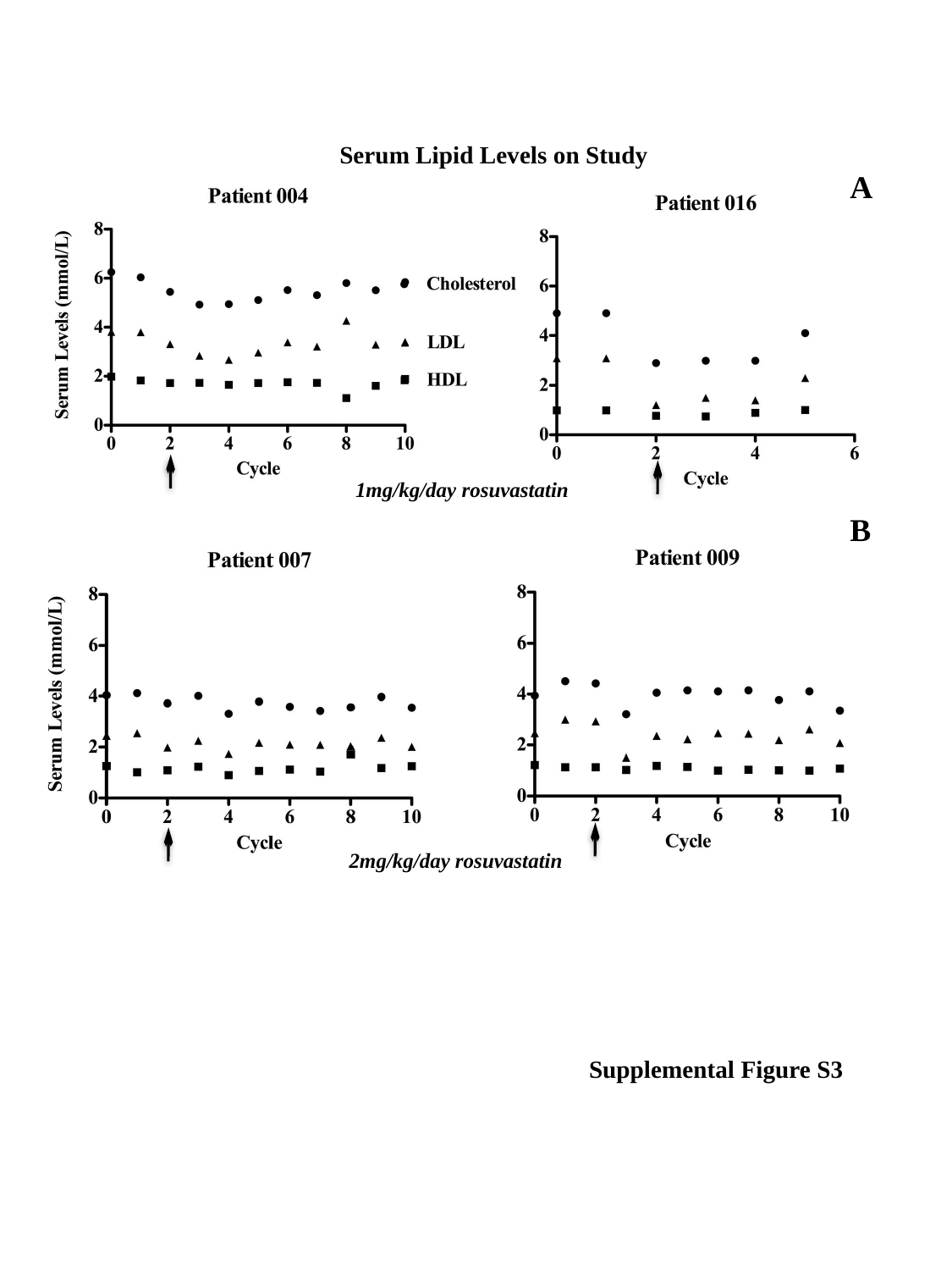

Serum Lipid Levels on Study
A
B
1mg/kg/day rosuvastatin
2mg/kg/day rosuvastatin
Supplemental Figure S3
